# Relationship and prognostic significance of SPARC and VEGF protein expression in colon cancer

**DOI:** 10.1186/1756-9966-29-71

**Published:** 2010-06-16

**Authors:** Jian-fang Liang, Hong-kun Wang, Hong Xiao, Ning Li, Cai-xia Cheng, Yu-ze Zhao, Yan-bo Ma, Jian-zhong Gao, Rui-bing Bai, Hui-xia Zheng

**Affiliations:** 1 Dept of Pathology, First Clinical Medical College, Shanxi Medical University, Taiyuan City, Shanxi, China; 2 Dept of Pediatrics, Shanxi Medical University, Taiyuan City, Shanxi, China; 3 Dept of Surgery, First Clinical Medical College, Shanxi Medical University, Taiyuan

## Abstract

**Background:**

SPARC (secreted protein, acidic and rich in cysteine) is closely related with the progress, invasion and metastasis of malignant tumor and angiogenesis.

**Methods:**

Using human colon adenocarcinoma tissues (hereinafter referred to as colon cancer) and their corresponding non-diseased colon from 114 patients' biopsies, the expression of SPARC and vascular endothelial growth factor (VEGF) were investigated by immunohistochemistry staining to assessment the relationship between SPARC and VEGF, as well as their prognostic significance in patients. Evaluation of VEGF expression level with the same tissues was used to establish the antigenic profiles, and the marker of CD34 staining was used as an indicator of microvessel density (MVD).

**Results:**

SPARC expression was mainly in the stromal cells surrounding the colon cancer, and was significant difference in those tissues with the lymph node metastasis and differentiation degree of tumor. Expression of SPARC was significantly correlated with the expression of VEGF and MVD in colon cancer tissues. Patients with low or absence expressing SPARC had significantly worse overall survival and disease-free survival in a Single Factor Analysis; Cox Regression Analysis, SPARC emerged as an overall survival and disease-free survival independent prognostic factor for colon cancer.

**Conclusion:**

The low expression or absence of stromal SPARC was an independent prognostic factor for poor prognosis of colon cancer. SPARC maybe involved in the regulation of anti-angiogenesis by which it may serve as a novel target for colon cancer treatment as well as a novel distinctive marker.

## Background

Colon cancer is a common malignant tumor of digestive tract. The incidence of colon cancer in China has increased in recent years. Angiogenesis (blood vessel growth) is a creitical process for tumor growth, invasion and metastasis. VEGF expression was closely related with biological behavior of colon cancer and significantly associated with high intratumoral microvessel density (MVD), and its over-expression in colon cancer tissue indicated poor prognosis [1]. Therefore, VEGF receptor inhibitors have been used to prevent the formation of blood vessels by arresting the growth of tumor cells. As a vascular endothelial marker, CD34 antigen by immunohistochemistry is used to evaluate the microvessel density (MVD) by reflecting the numbers of microvessel formation in the tumor tissues directly.

SPARC (Secreted Protein, Acidic and Rich in Cysteine; also known as BM-40 and osteonectin) was initially identified as osteonectin by Termine et al [2] as a bone-specific phosphoprotein that binds to collagen fibrils and hydroxyapatite at distinct sites. Recently, SPARC has generated considerable interests as a multi-faceted protein that belongs to a family of matricellular proteins. Differential expression of SPARC has been observed in various human cancers, and it is unclear why it has variable effects on tumor growth in different tissues [3]. For example, higher levels of SPARC expression have been reported in breast cancer, melanoma and glioblastomas. Yet, lower levels of SPARC expression have also been found in other types of cancers, such as ovarian and pancreatic. This pattern of decreased SPARC levels would suggest an inhibitory role for SPARC in tumor formation. In animal models of ovarian cancer [4,5], the absence of SPARC could de-repress the expressions of VEGF, by which to promote the angiogenic and metastatic potential of tumors. Other studies also found that, SPARC could bind with VEGF and decrease the capability of VEGF binding with its receptor, and resulted in the inhibition of endothelial cell proliferation [6-8].

The purpose of this study, was to explore the expression of SPARC and its relationship with angiogenesis, as well as the relationship between the other clinicopathological factors and prognosis with the expression of SPARC and VEGF. The results obtained in the current study were expected to provide an evidence for a novel molecular target therapy in colon cancer.

## Materials and methods

### Patients and tissue specimens

One hundred and fifty-three of colon cancers obtained between August 1999 and December 2003 were identified from our pathology files in Department of Pathology at the First Clinical Hospital of Shanxi Medical University, China. After review, 39 cases with synchronous other malignant tumors, familial adenomatous polyposis, colitis ulcerosa or Crohn's disease, using neoadjuvant therapy, lack of confirmatory surgical material, and/or clinical follow-up were excluded from this study. The remaining 114 cases were selected for SPARC, VEGF and CD34 staining.

A pair of tissue samples for each case was collected from the tumor tissues and their corresponding non-diseased colon. The protocol of this study was approved by our Institutional Review Board before all specimens were examined by the experienced pathologists. Histological examination was carried out on paraffin-embedded sections stained with hematoxylin & eosin (H&E). The patients were followed-up in a range of 4-110 months (median = 53 months), the mean survival time was 99.0 months and the five-year survival rate was 76.0%, median survival time was 81.7 months. Seventy two of these patients were found to be recurrence or metastasis with the metastatic sites of lymph nodes, stomach, spleen, liver, pancreas, ovary, cervix and bladder, and forty two cases died during the follow-up period. Other clinical and pathologic parameters were obtained from the pathological reports, including tumor differentiation, lymphocytic infiltration in the tumor interstitial and the TNM stage, and all of these data were reviewed and confirmed by the pathologists in our department (Table 1).

**Table 1:** Clinicopathologic characteristics of the colon cancer patients

Parameters	No. of patients(%)	Parameters	No. of patients(%)
Age (median, 59 years)		N2	13(11.4)
< 59	48(42.1)	Recurrence/distant metastasis	
≥ 59	66(57.9)	Yes	23(20.0)
Gender		No	91(79.8)
Men	54(47.4)	L/infiltration^a^	
Women	60(52.6)	Yes	41(36.0)
Tumor size(average 5.0)		No	73(64.0)
< 5.0	52 (45.6)	depth of invasion	
≥ 5.0	62(54.4)	T2	15(13.2)
Localization		T3	88(77.2)
colon ascendens	27(23.7)	T4	11 (9.6)
flexura hepatica	22(19.3)	Distant metastasis	
colon transversum	6(5.3)	M0	102(89.5)
flexura lienalis	8(7.0)	M1	12 (10.5)
colon descendens	6(5.3)	TNM staging	
colon sigmoideum	45 (39.5)	I	11(9.6)
Tumor differentiation		II	47(41.2)
low	16(14.0)	III	44(38.6)
moderate	68(59.6)	IV	12(10.5)
high	30(26.3)	Clinical outcome	
Lymph node metastasis		Disease free	72(63.2)
N0	65(57.0)	Metastasis or recurrence	72(63.2)
N1	36(31.6)	Death	42(36.8)

a lymphocytic infiltration in the tumor interstitial

Using WHO-OMS, IARC classification standard for colon cancer: well-differentiated adencarcinoma, > 95% glandular structure in tumor; moderately differentiated adencarcinoma, 50%~95% glandular structure in tumor; poorly differentiated adencarcinoma, 5%~50% glandular structure in tumor; anaplastic carcinoma < 5% glandular structure in tumor.

### Immunohistochemical (IHC) staining and scoring

Sections (4 μm) from the paraffin-embedded, formalin-fixed archival colon tissues were fixed on the charged slides for immunohistochemical analysis using non-biotin detection system (EnVision, Anti-Mouse/Rabbit-HRP, DAKO). Primary mouse monoclonal antibodies to SPARC (clone PP16, dilution 1:100), VEGF (clone C-1, dilution, 1:100) and CD34 (clone 43A1, dilution 1:150) (Santa Cruz, California, USA) were used in the study. All slides were deparaffinized with xylene and rehydrated through graded ethanol ending with distilled water. Then endogenous peroxidase was blocked by 3% hydrogen peroxide for 15 minutes. Sections for SPARC, VEGF and CD34 for immunohistochemical were subjected to microwave antigen retrieval with 0.1M citrate buffer (pH 6.0) at 98°C for 10 minutes, then were incubated overnight at 4°C in a humidified chamber, followed by EnVision detection incubated for 30 minutes at room temperature (RT). The staining were visualized by incubating with 3,3'-diaminobenzidine for 5 minutes at RT, then counterstained with hematoxylin. Negative (omission of primary antibody) and positive controls (paraffin sections of clone cancer) were run in parallel.

The intensity of immunostaining for SPARC was reviewed and scored according to the location of cytoplasmic with or without positive nucleus and results are presented by two independent observers without knowledge of the clinicopathological outcomes of the patients. The proportion of cells with SPARC expression was rated as follows [9-11]: 1 point, < 5% positive tumor cells; 2 points, 5~25% positive cells; 3 points, 26~75% positive cells; and 4 points, > 75% positive cells, and the intensity of staining varied from weak to strong. The intensity was classified as a scale of 0 (no staining), 1 (weak staining, light yellow), 2 (moderate staining, yellowish brown), and 3 (strong staining, brown). The specimens were attributed to four groups, according to their overall score: Absent expression, when < 5% of cells stained positive, regardless of intensity; weak expression, a total of 3 points; moderate expression, 4-5 points; and strong expression, 6-7 points. For statistical purpose, tumor cells were then scored according to a two-scale system: tumors with absent or weak expression was low reactivity, and with moderate to strong expression was high reactivity. The assessment of association of SPARC with other parameters using SPARC is either evaluated with a categorical variable (low reactivity vs. high reactivity) or a continuous variable (the percentage of SPARC-positive cells within a sample).

The staining results of VEGF were scored according to the percentage of cytoplasmic and/or membrane specific positive tumor cells. VEGF staining was reported as four grades, "-", positive staining in less than 5% of tumor cells; "1+" between 5% and 25%; "2+", between 26% and 50%; and "3+", more than 50%. The grades with less than 2+ were considered as low reactivity for VEGF, otherwise as high reactivity.

### Evaluation of microvessel density

Microvessels were identified by immunostaining endothelial cells with the mouse anti-human monoclonal antibody CD34. Microvessel density (MVD) was assessed according to the international consensus [12]. The entire section was scanned systematically at low magnification (× 100) in order to identify the most intense areas of neovascularization ("hotspots") within the tumor. After five hotspots areas with the highest number of capillaries and small venules were identified, microvessels were counted at high power magnification (× 400), and the average of count in five fields was calculated. MVD was quoted as a continuous variable [13,14].

### Statistical analysis

The Chi-square test or Fisher's exact probability test for proportion was used to analyze the relationship between SPARC and VEGF expression, and clinicopathologic characteristics. One-way ANOVA test and Linear regression analysis was used to assess the correlations among the continuous variables. Spearman rank correlation coefficient test analysis was performed to examine the correlations among different variables.. Survival curves were plotted by the Kaplan-Meier method, and compared by the log-rank test. To identify independent prognostic factors, including cancer recurrence, distant metastasis or death from disease, the Cox regression analysis was performed with the endpoints for disease-free survival (DFS) and overall survival (OS), respectively. A *P*-value of less than 0.05 was considered statistically significance. SPSS 11.5 was used for the statistical analysis.

## Results

### Expression of SPARC, VEGF, and CD34 in colon cancer and normal colon mucosa tissue

**Figure 1:**
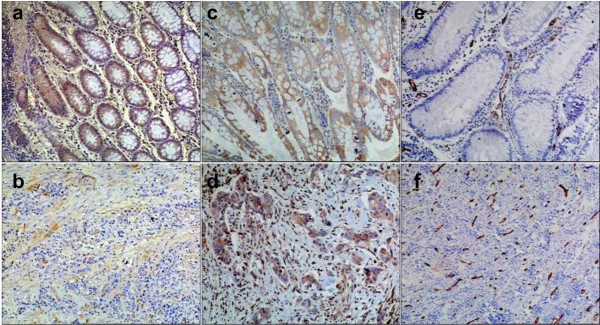
**Expression of SPARC, VEGF and CD34 in colon cancer and normal colon mucosa by Immunohistochemical staining**. a, c and e. SPARC, VEGF and CD34 expression in normal colon mucosa away from the colon cancer tissues; b. SPARC expression in MSC of colon cancer; d and f. VEGF and CD34 expression in colon cancer.

Expression of SPARC protein was determined by immunohistochemistry staining in 114 cases of paraffin-embedded colon cancer tissues and their corresponding non-diseased colon tissue. SPARC was mainly localized in the cytoplasm and was detected in the normal colonic epithelial cells (Fig 1a), the colon cancer cells and the mesenchymal and stromal cells (MSC) of colon cancer (Fig 1b). In this study, the degree of the expression of SPARC showed that 81 cases (71.1%) with low reactivity and 33 cases (28.9%) with high reactivity in tumor cells, 61 cases (53.5%) with low reactivity and 53 cases (46.5%) with high reactivity in the MSC surrounding the tumor, and 84 cases (73.7%) with low reactivity and 30 cases (26.3%) with high reactivity in the normal colon mucosa tissue, respectively. SPARC expression was no significant difference between the reactivity in tumor cells and in their corresponding non-diseased colon mucosa (*P *> 0.05), but was statistically significant difference between that in MSC and in tumor cells (*P *< 0.05), and between that in MSC and normal mucosa in colon tissue (*P *< 0.05), respectively.

The rate of positive VEGF expression was 72.8% in colon cancer cells and 47.4% in normal mucosal epithelical cells (Fig 1c, d) respectively, with a significant difference between them (*P *< 0.05).

CD34 was used to mark vascular endothelial cell or endothelial cell clustering around the tumors for MVD. The mean value of MVD was 11.60 ± 5.68 in all cases of the colon cancer, and MVD in tumor cells nest was significantly higher than that in the surrounding normal tissue (*P *< 0.05, Fig 1e, f).

### SPARC and VEGF protein expression vs. the MVD and the clinicopathological parameters

SPARC expression in colon cancer cells was no significant difference determined with clinicopathological parameters (*P *> 0.05), but SPARC expression in MSC was (1) significantly negative related to the differentiation of tumor (*P *< 0.05, r = -0.175); (2) statistically significant difference with lymph node metastasis (*P *< 0.05); and (3) no significant difference with the patients age, sex, tumor size, tumor location, lymphatic infiltration, and TNM staging (*P *> 0.05) (Table 2).

**Table 2:** Relationship of SPARC expression in colon cancer tissues with clinicopathological parameters

		Tumors cell					MSC				
Parameters		low reactivity high reactivity				*P *value	low reactivity high reactivity				*P *value
					
		n	%	n	%		n	%	n	%	
Age^a^						0.379					0.904
< 59	48	32	66.7	16	33.3		26	54.2	22	45.8	
≥ 59	66	49	74.2	17	25.8		35	53.0	31	47.0	
Gender						0.276					0.276
men	54	41	75.9	13	24.1		26	48.1	28	51.9	
women	60	40	66.7	20	33.3		35	58.3	25	41.7	
Tumor size^b^						0.222					0.658
< 5.0	52	34	65.4	18	34.6		29	55.8	23	44.2	
≥ 5.0	62	47	75.8	15	24.2		32	51.6	30	48.4	
Localization						0.140					0.926
colon ascendens	27	22	81.5	5	18.5		14	51.9	13	48.1	
flexura hepatica	22	17	77.3	5	22.7		12	54.5	10	45.5	
colon transversum	6	6	100	0	0		3	50.0	3	50.0	
flexura lienalis	8	6	75.0	2	25.0		3	37.5	5	62.5	
colon descendens	6	3	50.0	3	50.0		4	66.7	2	33.3	
colon sigmoideum	45	27	60.0	18	40.0		25	55.6	20	44.4	
Tumor differentiation						0.930					0.046
low	16	12	75.0	4	25.0		4	25.0	12	75.0	
moderate	68	48	70.6	20	29.1		39	57.4	29	42.6	
high	30	21	70.0	9	30.0		18	60.0	12	40.0	
Lymph node metastasis						0.462					0.013
N0	65	44	67.7	21	32.3		28	43.1	37	56.9	
N1	36	26	72.2	10	27.8		22	61.1	14	38.9	
N2	13	11	84.6	2	15.4		11	84.6	2	15.4	
R/DM^c^						0.490					0.746
Yes	23	15	65.2	8	34.8		13	56.5	10	43.5	
No	91	66	72.5	25	27.5		48	52.7	43	47.3	
L/infiltration^d^						0.626					0.678
Yes	41	28	68.3	13	21.7		23	56.1	18	43.9	
No	73	53	72.6	20	27.4		38	52.1	35	47.9	
depth of invasion						0.459					0.850
T2	15	12	80.0	3	20.0		8	53.3	7	46.7	
T3	88	60	68.2	28	31.8		48	54.5	40	45.5	
T4	11	9	81.8	2	18.2		5	45.5	6	54.5	
Distant metastasis						0.504					0.797
M0	102	71	69.6	31	30.4		55	53.9	47	46.1	
M1	12	10	83.3	2	16.7		6	50.0	6	50.0	
TNM staging						0.431					0.297
I	11	9	81.8	2	22.2		5	45.5	6	54.5	
II	47	30	63.8	17	36.2		21	44.7	26	55.3	
III	44	32	72.7	12	27.3		28	63.6	16	36.4	
IV	12	10	83.3	2	16.7		7	58.3	5	41.7	

a median, 59 years; b mean, 5.0 cm; c R/DM-Recurrence/distant metastasis;

d lymphocytic infiltration in the tumor interstitial

VEGF expression was statistically significant difference with lymph node metastasis, and was significantly correlated with TNM staging (*P *< 0.05, r = 0.302) (Table 3). The average MVD around the tumor nest had no significant difference with clinical pathological parameters (*P *> 0.05) (Table 3).

**Table 3:** Relationship of VEGF expression and MVD with clinicopathologic parameters and SPARC expression

Parameters		VEGF				*P *value	MVD (CD34)	*P *value
					
		(-)	(1+)	(2+)	(3+)		(mean ± S.D.)	(ANOVA)
Total	114	31	27	22	34		11.60 ± 5.68	
Age						0.612		0.319
< 59	48	11	10	10	17		12.23 ± 6.19	
≥ 59	66	20	17	12	17		11.15 ± 5.28	
Tumor differentiation						0.112		0.952
low	16	6	2	3	5		11.24 ± 7.30	
moderate	68	16	18	9	25		11.72 ± 5.30	
high	30	9	7	10	4		11.53 ± 5.75	
Lymph node metastasis						0.001		0.879
N0	65	23	20	13	9		11.80 ± 5.54	
N1	36	7	6	7	16		11.20 ± 6.74	
N2	13	1	1	2	9		11.74 ± 2.59	
depth of invasion						0.601		0.281
T2	15	5	3	4	3		11.28 ± 5.63	
T3	88	24	21	14	29		11.33 ± 5.66	
T4	11	2	3	4	2		14.20 ± 5.72	
TNM staging						0.002		0.295
I	11	4	3	3	1		12.00 ± 6.00	
II	47	17	15	8	7		10.99 ± 4.70	
III	44	8	6	6	24		11.04 ± 6.26	
IV	12	2	3	5	2		14.26 ± 5.46	
SPARC in MSC						0.0001		0.027
low reactivity	61	17	6	13	25		12.69 ± 5.71	
high reactivity	53	14	21	9	9		10.34 ± 5.43	

### Correlation analysis of SPARC expression in MSC with VEGF expression and MVD

**Figure 2:**
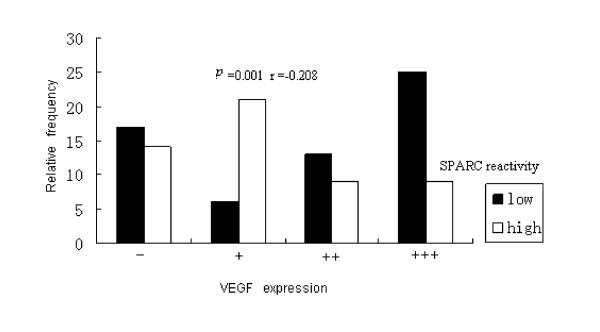
**Correlation analysis of SPARC expression in MSC and VEGF expression in colon cancer**.

**Figure 3:**
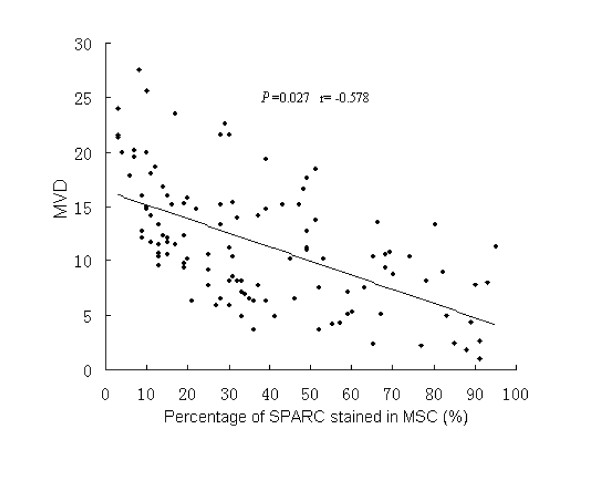
**Linear regression analysis of the percentage of SPARC stained in MSC with MVD**.

Using Spearman rank correlation analysis, SPARC expression in MSC was negative significantly related with VEGF in colon cancer tissue (*P *< 0.05, r = -0.208) (Table 3, Fig 2). Linear regression analysis of SPARC-positive percentage of individual cases in MSC showed significant correlation with MVD in these human colon cancer specimens (*P *< 0.05, r = -0.578) (Table 3, Fig 3).

### Survival analysis

**Figure 4:**
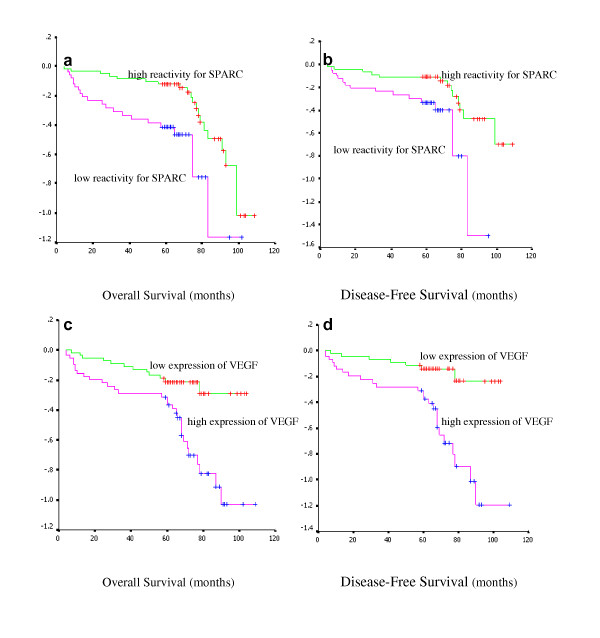
**Kaplan-Meier survival curve for SPARC and VEGF protein expression in colon cancer patients**. Comparison of overall as well as disease-free survival between the groups of patients with low and high SPARC and VEGF protein expression.

Kaplan-Meier analysis and the log-rank test were used to evaluate the effects of the SPARC and VEGF expression on survival. There was a significantly unexpected influence on SPARC expression in MSC between the group of low reactivity and high reactivity on both OS (*P *< 0.05) and DFS (*P *< 0.05) of the patients (Fig 4a, b). On the contrary, patients with high reactivity of VEGF have poor prognosis than those with low reactivity for either the overall survival (*P *< 0.05) or disease-free survival (*P *< 0.05) (Fig 4c, d).

In this study, the multivariate survival analysis were used, including SPARC expression level in MSC, VEGF expression level, MVD, tumor differentiation, lymph node metastasis, lymphoid infiltration, invasion depth, distant metastasis and TNM staging to test the independent effects of SPARC on survival (Table 4). The results indicated that SPARC expression (*P *< 0.05), VEGF expression (*P *< 0.05) and TNM staging (*P *< 0.05) were independent prognostic factors for OS, and SPARC expression (Table 5) was also an independent prognostic factor of DFS (*P *< 0.05).

**Table 4:** OS analysis of different prognostic factors in patients with colon cancer by Cox Regression Analysis

Parameters	Regression Coefficient	Standard Error	Wald	Relative Risk	95%CI		*P *Value
						
					lower	upper	
Tumor differentiation	0.076	0.280	0.074	1.079	0.623	1.869	0.785
Lymph node metastasis	-0.174	0.363	0.230	0.840	0.412	1.712	0.632
L/infiltration^a^	-0.012	0.384	0.001	0.989	0.466	2.097	0.976
depth of invasion	-0.344	0.431	0.639	0.709	0.305	1.649	0.424
Distant metastasis	-0.205	0.459	0.200	0.815	0.331	2.003	0.655
TNM	0.959	0.363	6.972	2.609	1.280	5.316	0.008
SPARC	0.999	0.367	7.431	2.717	1.324	5.574	0.006
VEGF	-0.311	0.153	4.136	0.733	0.543	0.989	0.042
MVD	0.026	0.028	0.887	1.027	0.972	1.085	0.346

a lymphocytic infiltration in the tumor interstitial

**Table 5:** DFS analysis of different prognostic factors in patients with colon cancer by Cox Regression Analysis

Parameters	Regression Coefficient	Standard Error	Wald	Relative Risk	95%CI		*P *Value
						
					lower	upper	
Tumor differentiation	0.157	0.355	0.196	1.170	0.583	2.348	0.658
Lymph node metastasis	-0.165	0.622	0.070	0.848	0.250	2.873	0.792
L/infiltration^a^	-0.101	0.431	0.054	0.904	0.388	2.106	0.816
depth of invasion	-1.021	0.611	2.792	0.360	0.109	1.193	0.095
TNM staging	0.881	0.565	2.433	2.413	0.798	7.298	0.119
SPARC	0.957	0.441	4.695	2.603	1.096	6.184	0.030
VEGF	-0.242	0.192	1.598	0.785	0.539	1.143	0.206
MVD	0.039	0.031	1.607	1.040	0.979	1.104	0.205

a lymphocytic infiltration in the tumor interstitial

## Discussion

The development, invasion and metastasis of malignant tumors depend on a pathological environment which provides sufficient nutrients to promote the neovascularization and complex cell-cell and cell-matrix interactions. On the other hand, tumor cells can produce a number of soluble proteins into the adjacent extracellular matrix (ECM) organization to facilitate the communication between tumor cells and their environment by stimulating the tumor cell growth.

SPARC as a member of the family of matricellular proteins, is a calcium-binding protein. SPARC is not only binding on the several resident proteins of the ECM, but also is competitively binding on the cell membrane surface growth factor receptor to modulate growth factor signaling [2]. SPARC has profound influence on cancer progression [15]. As a secreted acidic and cysteine-enriched protein in the ECM, SPARC inhibits the proliferation of different cell types and modulates tumor cell aggressive features. This apparent paradox might result either from the biochemical properties of the different SPARC sources (endogenous or exogenous) or from differential responses of malignant and stromal cells to SPARC [16]. In cancer, the expression pattern of SPARC is variable depending on the tumor types. For example, a strong cytoplasmic SPARC expression was found in stromal cells surrounding malignant tissues in breast cancer, but was absent in stromal cells of normal breast tissues [17,18], and SPARC expression in the surrounding stromal of breast cancer was significantly higher than tumor cells [19,20]. Similar observations were made in prostate cancer [21], bladder cancer [22], non-small cell lung cancer [23] and ovarian cancer [24].

There are not only the differences in the pattern of SPARC expression within tumors and the stroma surrounding malignant tissues, but also the differential clinical outcomes of SPARC expression in a variety of tumors. Watkins, et al. [25] showed that high levels of SPARC expression in tumor cells negatively correlated with the overall survival of patients in breast cancer, but was unrelated to the disease-free survival. Recent studies have shown that over-expression of SPARC in the surrounding stromal of breast cancer was related with the better prognosis of patients [19,20]. However, the increased SPARC expression in prostate cancer, bladder cancer and non-small cell lung cancer indicated a higher malignancy and invasion of tumors with poor prognosis. In contrast, in ovarian cancer, elevated SPARC expression inhibited the invasion and metastasis of tumor cells [4].

Recently, the role of SPARC expression in colon cancer was concerned greatly. To investigate if SPARC promotes or inhibits the invasion and metastasis of tumor, the expression level of SPARC in human colon cancer tissues and their corresponding non-diseased colon by immunohistochemical method in the current study. The results in our study showed that SPARC expression in MSC was significantly higher than that in cancer cells and in normal mucosa tissues, and only SPARC expression in MSC was significantly different with clinicopathological parameters including tumor differentiation and lymph node metastasis. Our results also showed that SPARC expression was mainly in MSC and decreased in colon cancer tissue, which indicated that SPARC might inhibit the invasion and metastasis of tumor during colon cancer development. Others considered that this suppression might be related to the tumor growth, and SPARC had an antiproliferative function through modulating cell cycle regulatory proteins or growth factors [26]. Similar results have been reported in lung cancer and pancreatic cancer [27,28].

SPARC has been found to act as an angiogenesis inhibitor by regulating the activities of growth factors like VEGF and platelet-derived growth factor [29-32]. While regulating VEGF, SPARC can bind to VEGF through EF-arm of the FS and EC areas to inhibit VEGF-stimulated proliferation of endothelial cells [7,8,33]. The role of slowing and terminating the tumor growth with SPARC by inhibiting the synthesis and secretion of VEGF has been reported in glioma [34]. Similarly, Chlenski et al. [35] found that SPARC is an inhibitor of angiogenesis in Schwann cells. They showed that MVD value of SPARC-treating group was significantly lower than non-treated control group and demonstrated that purified SPARC potently inhibited neuroblastoma growth and angiogenesis in vivo.

In the current study, from the expression pattern of SPARC and VEGF, we found that VEGF and SPARC were mainly expressed in tumor cells and MSC, respectively. The expression of the angiogenic factor VEGF and the intratumoral vascular density were apparently not related to the production of SPARC in MSC, however, high levels of SPARC in MSC was significantly negative related with VEGF expression and MVD counts. In addition, our results showed that VEGF was significantly different with lymph node metastasis and TNM staging. VEGF expression was up-regulated in colon cancer along with the decreased expression of SPARC. All of these results suggest that SPARC may inhibit VEGF expression during the process of new blood vessel growth by which indirectly control the development, growth, invasion and metastasis of tumor cells in colon cancer.

We also analyzed the relationships of SPARC and VEGF expression with clinical prognosis in this study. The results showed that patients with low expression of VEGF were survival longer than those with high expression for overall or disease-free survival evaluated by Kaplan-Meier analysis. Similar results reported by Des et al. [1]. They investigated 27 kinds of VEGF expression in colorectal carcinoma using Meta analysis, and found that high levels of VEGF expression were related with unfavorable prognoses. Moreover, they revealed that VEGF was a more effective marker than MVD for prediction of overall survival in patients.

We believe that increased expression of VEGF correlates with decreased SPARC expression. Reduction of SPARC may up-regulate the expression of VEGF, causing the subsequent MVD increase in tumors and resulting in a poor clinical outcome. Analysis for overall and disease-free survival showed that patients with low or absence of SPARC expression displayed a poor prognosis, when compared with patients with higher SPARC expression. Therefore, it may support an hypothesis that SPARC potentially regulates the expression of angiogenesis factor VEGF during colon cancer development, by regulating indirectly the formation of blood capillary, to impact the clinical prognosis of patients.

Clinicopathological parameters including lymph node metastasis, lymphocytic infiltration in the tumor interstitial, depth of invasion, distant metastasis, TNM staging, may effect on the prognosis of patients, the expression of SPARC and VEGF, and MVD value, with multivariable models. The results of the analysis of the cinicopathological parameters showed that SPARC expression influences independently overall and disease-free survival of patients with colon cancer and is an independent prognostic factor for colon cancer. Moreover, TNM staging and VEGF expression were also independent negative prognostic factors on overall survival. Although lymph node metastasis is commonly considered as an important prognostic factor for colon cancer, the results in this study did not show that lymph node metastasis correlate with overall and disease-free survival, which may be related to race itself and the relevant regional. Further investigation of the effects of these factors should be taken for the reasonable and reliable evidence in the future.

Recent studies, both *in vitro *and *in vivo*, have found the role of exogenous SPARC on tumor cell biological behaviors. For example, in ovarian cancer cells [36], exogenous exposure to SPARC resulted in the enhanced apoptosis, whereas endogenous absence of it diminished apoptosis. In melanoma cells and colorectal cancer cells, exogenous addition of SPARC significantly inhibited the cell proliferation and enhanced chemosensitivity of tumor cells that had become resistant to chemotherapy when compared with those tumor cells that were deficient in endogenous SPARC [15].

With the results of current study, we speculate that endogenous expression of SPARC may inhibit VEGF-stimulated capacity of angiogenesis in the development process of colon cancer. The possible reason for the low expression or absence of SPARC in high malignant colon cancer tissue is that either endogenous SPARC expression is down-regulated or its secretion is arrested by other factors. Based on this hypothesis, insufficient SPARC might inhibit the production of blood capillary, which leads to the unlimited growth of tumors.

## Conclusions

In summary, the expression of SPARC protein can emerge in tumor cells and MSC of colon cancer, but mainly in MSC. SPARC expression in MSC positively correlates with tumor differentiation and lymph node metastasis and may be involved in regulation of production of angiogenesis factor VEGF. It is believed that inhibition of SPARC expression is associated with the tumor progress and invasion process of colon cancer. In addition, low expression or absence of SPARC protein in MSC can be considered as an important independent unfavourable prognostic factor of colon cancer. Importantly, the regulatory mechanism points to the possibility that SPARC-based gene and protein therapy can be used with current therapeutic modalities to affect tumor regression in advanced colon cancer refractory to therapy and will be a meaningful frame of reference of molecular target therapy of tumor.

## Abbreviations

SPARC: secreted protein, acidic and rich in cysteine; VEGF: vascular endothelial growth factor; MVD: microvessel density; H&E: hematoxylin & eosin; RT: room temperature; DFS: disease-free survival; OS: overall survival; MSC: mesenchymal and stromal cells

## Competing interests

The authors declare that they have no competing interests.

## Authors' contributions

JFL, HX and HXZ were equally involved in the design of the study and drafted the manuscript. HKW was involved in the design of the study, patient recruitment, management of the patients, statistical analysis and drafted the manuscript. JZG and RBB carried out most of the experiments. CXC, NL, YBM and YZZ participated in data organization and manuscript drafting. All authors read and approved the final manuscript.
